# Applying a new weaning index in ICU older patients

**DOI:** 10.1186/cc10183

**Published:** 2011-06-22

**Authors:** LM Azeredo, SN Nemer, JB Caldeira, B Guimarães, R Noé, CP Caldas, M Damasceno

**Affiliations:** 1 Hospital de Clínicas de Niterói, Niterói - RJ, Brazil

## Introduction

With the increase in life expectation, more admissions to hospital, use of mechanical ventilation (MV) and weaning trials in older patients have been observed.

## Objective

To evaluate the variables associated with successful weaning from mechanical ventilation in older patients.

## Methods

We evaluated a cohort from September 2004 to January 2008 with 479 patients. We excluded one patient aged under 18 years, 35 tracheostomized and 112 with neurologic diseases, resulting in 331 patients. Besides the conventional weaning indexes, we evaluated the performance of a new integrative weaning index (IWI). The study was approved by the Ethics Committee of Pedro Ernesto University Hospital (2206-CEP). The chances of successful weaning were investigated using relative risk and logistic regression. The Hosmer-Lemeshow goodness-of-fit test was used to calibrate and the C statistic was calculated to evaluate the association between predicted probabilities and observed proportions in the logistic regression model.

## Results

Prevalence of successful weaning in the sample was 83.7%. There was no difference in mortality of older and nonolder patients (*P *= 0.16), in the days of mechanical ventilation (*P *= 0.22) and days of weaning (*P *= 0.55). In older patients, the IWI was the only variable associated with respiratory weaning in this population (*P *< 0.0001). See Tables 1 to 5.

**Table 1:** Etiology and population

Etiology	Population (%)
COPD, *n *(%)	98 (29.6)
Pneumonia, *n *(%)	68 (20.54)
Postoperative, *n *(%)	63 (19.03)
Sepsis, *n *(%)	39 (11.78)
ARDS/ALI, *n *(%)	25 (7.55)
Trauma without brain injury, *n *(%)	11 (3.32)
Acute pulmonary edema, *n *(%)	10 (3.02)
Miscellaneous, *n *(%)	17 (5.13)
Total	331

ALI, acute lung injury; ARDS, acute respiratory distress syndrome; COPD, chronic obstructive pulmonary disease.

**Table 2:** Analysis of the outcome variables by sample and by age

Variable	Category	Total (%)	Age ≥70 (%)	Age <70 (%)	*P *value
Result	Success	277 (83.7)	125 (80.7)	152 (86.4)	0.16
	Failure	54 (16.3)	30 (19.4)	24 (13.6)	
	Death	17 (5.1)	15 (9.7)	2 (1.1)	0.002
Evolution	Discharge	277 (83.7)	125 (80.7)	152 (86.4)	
	Return	37 (11.2)	15 (9.7)	22 (12.5)	
Days of MV	Mean ± DP/median	9.1 ± 7.6/7	9.2 ± 8.6/6	9.1 ± 6.7/7	0.22
Days of weaning	Mean ± DP/median	2.7 ± 2.3/2	2.8 ± 2.6/2	2.6 ± 2.0/2	0.55
APACHE II	Mean ± DP/median	16.0 ± 5.6/15	16.9 ± 5.8/16	15.3 ± 5.2/14	0.009

Significant *P *< 0.05.

**Table 3:** Analysis of the respiratory variables according to the results by age

	Age ≥70				Age <70			
		
Variable	Success (%)	Failure (%)	RR	95% CI	Success (%)	Failure (%)	RR	95% CI
P/F ≥255	59.2	46.7	1.11	0.94 to 1.30	63.2	16.7	1.30	1.13 to 1.50
Cqst.rs ≥30	80.8	23.3	1.83	1.38 to 2.43	84.2	29.2	1.62	1.25 to 2.10
IWI ≥25.5	97.6	3.3	10.6	3.60 to 31.1	96.1	8.3	4.60	2.26 to 9.36
P 0.1 ≤3.1	78.4	36.7	1.53	1.19 to 1.97	77.0	20.8	1.48	1.21 to 1.81
f ≤29	72.8	33.3	1.43	1.16 to 1.77	68.4	20.8	1.33	1.14 to 1.56
Vt ≥320	76.8	23.3	1.67	1.31 to 2.14	73.7	29.2	1.34	1.13 to 1.60
f/Vt*P 0.1 ≤270	80.0	33.3	1.64	1.25 to 2.14	77.0	16.7	1.52	1.24 to 1.86
f/Vt ≤100	81.6	23.3	1.87	1.40 to 2.51	78.3	25.0	1.47	1.20 to 1.81

RR, relative risk. Significant *P *< 0.0001, except for P/F on age ≥70.

**Table 4:** Logistic regression to the success of weaning by age

Age	Significant Variable	Coefficient	SE	*P *value	RR	95% CI
≥70	Intercept	-2.2687	0.606	0.0002		
	IWI ≥25.5	7.0727	1.173	<0.0001	1,179.3	118 to 11,752
<70	Intercept	-3.1147	1.035	0.003		
	IWI ≥25.5	6.0547	1.187	<0.0001	426.1	41.6 to 4,364
	APACHE ≤17	3.4249	1.159	0.003	30.7	3.2 to 298

CI, confidence interval; RR, relative risk; SE, standard error of coefficient.

**Table 5:** Estimated probability of success according to the logistic model by age group

Age	IWI ≥25.5	APACHE ≤17	Estimated probability (%)	95% CI
≥70	No		9.4	3.06 to 25.4
	Yes		99.2	94.5 to 99.9
<70	No	No	4.3	0.58 to 25.2
	No	Yes	57.7	27.5 to 83.1
	Yes	Yes	95.0	81.8 to 98.8
	Yes	No	99.8	98.0 to 100.0

CI, confidence interval: lower limit (%) to upper limit (%).

## Conclusion

The IWI was the main independent variable in weaning of the older patient population, and it can contribute to this critical moment.

